# Exploring the transformative effects of calorie restriction on the lacrimal gland in adult mice

**DOI:** 10.1007/s11357-025-01748-w

**Published:** 2025-06-28

**Authors:** Olivier Mauduit, Prashant Kumar, Kaitlin K. Scholand, Emre Aksan, Laura Schaefer, Anmar Abu-Romman, Vanessa Delcroix, Zhiyuan Yu, Aude I. Sindikubwabo, Ron Korstanje, Helen P. Makarenkova, Cintia S. de Paiva

**Affiliations:** 1 Department of Molecular Medicine, The Scripps Research Institute, San Diego, CA USA; 2 Department of Ophthalmology, Ocular Surface Center, Cullen Eye Institute, Baylor College of Medicine, Houston, TX USA; 3 Department of Biosciences, Rice University, Houston, TX USA; 4 Department of Molecular Virology and Microbiology, Center of Metagenomics and Microbiome Research, Baylor College of Medicine, Houston, TX USA; 5 The Jackson Laboratory, Bar Harbor, ME USA

**Keywords:** Aging, Lacrimal gland, Dry eye, Calorie restriction

## Abstract

Advanced age is one of the most recognizable risk factors for dry eye. Dry eye disease affects millions worldwide and can result from age-related lacrimal gland dysfunction, which correlates with a decline in lacrimal gland secretory cell function and chronic inflammation. This study investigated the potential of calorie restriction to maintain lacrimal gland and ocular surface health. Adult female C57BL/6 J mice were subjected to a 40% calorie restriction for 4 months, starting at 6–7 months and continuing until 10–11 months. These mice were compared to controls fed ad libitum. Bulk RNA sequencing of lacrimal glands, conjunctiva, and cornea subjected to calorie restriction compared to ad libitum revealed significant differentially expressed genes (DEGs). Pathways enriched in the upregulated DEGs indicate enhanced circadian rhythm, secretory functions, and lipid metabolism. These findings were confirmed using individual qRT-PCR and western blotting. In contrast, pathways enriched in the downregulated DEGs were associated with immune cell activation, adaptive immune responses, extracellular matrix remodeling, and metalloproteinase activity. Histological sections of calorie-restricted lacrimal glands revealed reduced mononuclear cell infiltration and fewer positive cells for CD4, CD19, and MHC II than in ad libitum lacrimal glands. Calorie restriction also prevented age-related corneal barrier dysfunction and mitigated age-related conjunctival goblet cell loss, hallmarks of dry eye disease. These findings suggest that calorie restriction supports lacrimal gland and ocular surface health by reducing inflammation and extracellular matrix remodeling and by enhancing the lacrimal gland’s secretory function.

**Supplementary Information:**

The online version contains supplementary material available at 10.1007/s11357-025-01748-w.

## Introduction

According to the World Health Organization, aging of the human population is one of the most significant social transformations of the twenty-first century [1]. Aging is a complex biological process often accompanied by a decline in physiological function and tissue degeneration. One hallmark of aging is chronic, low-grade inflammation, called inflammaging [2], which is characterized by a systemic increase in the production of proinflammatory cytokines such as IL-1β, tumor necrosis factor (TNF), and IL-6. Notably, elevated serum levels of IL-6 and TNF have been identified as predictors of disability and mortality in octogenarians and centenarians [3–5]. Individual differences in the regulation of pro- and anti-inflammatory cytokines may play a critical role in determining the outcomes of age-related inflammatory responses in epithelial tissues and immune systems [6].

Dry eye disease is an inflammatory disorder of the ocular surface in which tear film instability and reduced tear production lead to hyperosmolar tissue damage [7]. Patients with dry eye disease have reduced quality of life due to chronic pain, irritation, and blurry vision [8]. Dry eye disease increases in prevalence with aging, highlighting the need for therapeutics that address the inflammation that accompanies aging [9].

Inflammation and dysfunction of the lacrimal gland (LG) are major contributors to dry eye disease in aging [10–15]. The LG is an exocrine tubuloacinar gland that secretes the aqueous layer of the tear film onto the ocular surface. Dysfunction in tear secretion can result in visual disturbances, eye irritation, and, in severe cases, corneal perforation. Several studies have shown that lifestyle changes, such as exercise, improved nutrition, calorie restriction (CR), and dietary supplements, positively affect longevity and reduce age-related inflammatory diseases, demonstrating that age-related changes can improve health [6, 16–20]. CR is a well-recognized intervention that increases longevity in rodents and large animals [21]; however, the effects of CR on age-related dry eye disease are poorly understood. Only two publications to date have investigated CR effects on LG. One study showed that CR can improve tear volume, decrease the corneal fluorescein staining score, and improve oxidative stress immunoreactivity in 6- to 12-month (M) rat LGs [22]. In the second study, female rats that received 50% CR from 14 to 20 M improved LG structure and decreased IL-6 and TNF levels compared to the age-matched group fed ad libitum (AL) [23]. While these studies have reported a positive effect of CR on the LG, these were descriptive studies without a potential molecular mechanism. Therefore, there is a need to investigate further how CR affects the LG and ocular surface homeostasis.

This study comprehensively examined the effects of CR on LGs and eyes of middle-aged mice (6–7 to 10–11 M), representing an early stage of aging. Using bulk RNA sequencing, we demonstrated that CR initiated at this stage significantly reshapes the LG, cornea, and conjunctiva transcriptome and mitigates the aging-related dry eye phenotype by improving corneal barrier function, decreasing mononuclear infiltrates to the LG, and protecting against age-related goblet cell loss. CR reduced inflammation while enhancing LG secretory activity and metabolic processes.

## Material and methods

### Animals and establishing CR

The Institutional Animal Care and Use Committee at Baylor College of Medicine, The Scripps Research Institute, and Jackson Laboratories approved all animal experiments. In addition, all studies adhered to the Association for Research in Vision and Ophthalmology for the Use of Animals in Ophthalmic and Vision Research and the NIH Guide for the Care and Use of Laboratory Animals [24].

Given that dry eye is more prevalent in women than men [25, 26], and that aged male mice do not develop corneal barrier disruption, a hallmark of dry eye [27], this study exclusively used female mice. Female C57BL/6 J mice were purchased from The Jackson Laboratory (Bar Harbor, Maine) or aged in-house. These mice were used for the CR experiments. Additional aged mice were received from the National Institute on Aging and used at 24 months or older. Two different cohorts of mice were used for the dietary restriction. For cohort 1, inbred female C57BL/6 J mice were fed a standard mouse chow diet (5K0G, Lab Diet, Fort Worth, Texas) at Jackson Laboratory. CR was established gradually as previously described [28]. Mice were co-housed up to five mice per pen. For the second cohort, inbred female C57BL/6 J received a complete 5VR diet (ad libitum group (AL), LabDiet) while mice on calorie restriction received the 5VR diet with a 40% increase in vitamins and micronutrients (diet 5GBC, LabDiet). The second study was performed at the Ocular Surface Center, Department of Ophthalmology, Baylor College of Medicine (Houston, Texas). Mice were co-housed up to five mice per pen. A group of C57BL/6 J 6–7 M mice was randomized to receive 40% CR or fed AL for 4 months (*n* = 15–16/group). Food consumption was calculated per cage 2 weeks before the CR. CR was performed in a stepwise manner: mice received 10% less food in the first week, followed by 20% less food in the second week and 40% less food in the third week. Mice were weighed at the start of the study and then monitored weekly. CR mice were fed daily at 5:00 PM, just before the start of the night cycle.

The sample sizes and age groups were as follows: young (2–3 M, *n* = 14), middle-aged (12–14 M, *n* = 16), and advanced-aged (24 M, *n* = 8). The 24-M mice were not subjected to CR due to their fragility but were used to investigate aging-related changes.

Multiple tissues were collected from each mouse to reduce animal use. The final sample size per endpoint is shown in the figure legends.

### RNA isolation

Corneal epithelial cells were collected by scraping and the conjunctiva was surgically excised. Exorbital LGs were harvested. Samples were added immediately to the Qiagen lysis buffer and stored at −80 °C until ready to be extracted. Total RNA was isolated using a QIAGEN RNeasy Plus Mini RNA isolation kit (Qiagen; Hilden, Germany) following the manufacturer’s protocol. After isolation, RNA concentration was measured, and the RNA quality was assessed by Bioanalyzer. Only samples with an RNA integrity number > 7 were selected. Total RNA was used for bulk RNA sequencing or in individual qPCR reactions.

### RNA sequencing

A double-stranded DNA library was created at Baylor College of Medicine’s Genomics and RNA Profiling Core. Briefly, using 100 ng of total RNA (measured by PicoGreen), an oligo(dT) primer containing an Illumina-compatible sequence at its 5′ end was hybridized to the RNA, and reverse transcription was performed using a Lexogen kit. Second-strand synthesis was initiated by a random primer containing an Illumina-compatible linker sequence at its 5′ end. The purified double-stranded library was then amplified and purified. The resulting libraries were quantitated using Qubit 2.0 (Thermo Fisher), and fragment size was assessed with the Agilent Bioanalyzer (Agilent Technologies, Santa Clara, California). A qPCR quantitation was performed on the libraries to determine the concentration of adapter-ligated fragments using Applied Biosystems ViiA7 ™ Real-Time PCR System and a KAPA Library Quant Kit (Waltham, Massachusetts). All samples were pooled equimolarly, re-quantitated by qPCR, and reassessed on the bioanalyzer. Using the concentration from the ViiA7 ™ qPCR machine above, 1.8 pM of the equimolarly pooled library was loaded onto NextSeq 500 high-output flow cell (Illumina, San Diego, California). PhiX Control v3 adapter-ligated library (Illumina) was spiked in at 1% by weight to ensure balanced diversity and to monitor clustering and sequencing performance. A single-read 75-base pair cycle run was used to sequence the flow cell. An average of 21 million reads per sample was sequenced. The FastQ file generation was executed using Illumina’s cloud-based informatics platform, BaseSpace Sequencing Hub (Illumina).

FASTQ files generated by sequencing were analyzed using ROSALIND (https://rosalind.bio/), which features a HyperScale architecture developed by ROSALIND, Inc. (San Diego, California). Reads were trimmed using cutadapt [29]. Quality scores were assessed using FastQC. Reads were aligned to the Mus musculus genome build mm10 using STAR [30]. Individual sample reads were quantified using HTseq [31] and normalized via Relative Log Expression (RLE) using DESeq2 R library [32]. Read Distribution percentages, violin plots, identity heatmaps, and sample MDS plots were generated as part of the QC step using RSeQC [33]. DEseq2 was also used to calculate fold changes and *p*-values and perform optional covariate correction. The FASTQ files and processed data are available on GEO under accession number GSE281747 (lacrimal gland) and GSE298317 (cornea and conjunctiva).

From the list of DEGs identified by RNAseq, we submitted the lists of upregulated and downregulated DEGs to Metascape and performed a pathways enrichment analysis. Then, we selected the top 10 biological pathways in which we had at least 10 genes altered and the FDR inferior to 0.05. Bar plots of significantly altered pathways were generated using Metascape with default parameters [34].

### Real-time PCR

Individual reactions used total RNA from LG lysates. cDNA was synthesized using the Ready-To-Go You-Prime First-Strand kit (GE Healthcare, Chicago, Illinois). Real-time PCR was performed using specific TaqMan minor groove binder probes for potassium calcium-activated channel subfamily N member 4 (*Kcnn4*, Mm00464586), FXYD domain containing ion transport regulator 2 (*Fxyd2*, Mm00446358_m1), solute carrier family 12 member 2 (*Slc12a2*, Mm01265951), adiponectin (*Adipoq*, Mm04933656), lipopolysaccharide binding protein (*Lbp*, Mm00493139), adenosine A1 receptor (*Adora1*, Mm01308023), cytochrome P450 family 2 subfamily E member 1(*Cyp2e1*, Mm00491127), proline-rich protein BstNI subfamily 1 (*Prb1*, Mm03060478), IL-1β (*Il1b*; Mm00434228), major histocompatibility complex class II (*Ciita*, MHC II; Mm00482914), interferon-gamma (*Ifng*; Mm00801778), tumor necrosis factor (*Tnf;* Mm00443258), IL-12 (*Il12a*, Mm00434165_m1), CD4 (*Cd4*, Mm00442754), IL-18 (*Il18*, Mm00434225), absent in melanoma 2 (*Aim2*, Mm01295715), CD19 (*Cd19,* Mm00515420), CXCL13 (*Cxcl13*,Mm00444533), Glycam-1 (*Glycam1*, Mm00801716_m1), CXCR5 (*Cxcr5*, Mm00432086), CCR7 (*Ccr7,* Mm00432608), and TaqMan Universal PCR Master Mix AmpErase UNG in a commercial thermocycling system (StepOnePlus Real-Time PCR System Applied Biosystems/Thermo Fisher Scientific, Foster City, California), according to the manufacturer’s recommendations. The hypoxanthine phosphoribosyltransferase 1 (*Hprt1*; Mm00446968) gene was used as an endogenous reference for each reaction. The quantitative PCR results were analyzed by the comparative Ct method and were normalized by the Ct value of *Hprt1*. The young group served as calibrators in the time course experiment comparing 2 M, 12, and 24 M, while the AL group was the calibrator in the CR experiment.

### Western blotting

LGs from AL and CR mice were harvested and lysed in RIPA lysis buffer (Thermo Fisher, Waltham, Massachusetts) plus protease inhibitors cocktail (SIGMA, St. Louis, Missouri). Protein concentration was measured using the micro-BCA protein assay kit (Thermo Fisher). LG lysates (40 µg) were resuspended in SDS sample buffer, boiled for 5 min, and analyzed on 4–15% mini-protean TGX stain-free gels (Bio-Rad, Hercules, California). The proteins were electrophoretically transferred to polyvinylidene difluoride membranes (Bio-Rad). The blots were probed with an anti-LBP polyclonal antibody (Proteintech, Rosemont, Illinois), anti-adiponectin (Proteintech), anti-IL-18 (Thermo Fisher Scientific), anti-IL-1β (Cell Signaling Technology, Danvers, Massachusetts), or an anti-actin antibody (SIGMA) overnight at 4 °C. The blots were washed extensively with a solution containing 50 mM Tris, pH 8.0, 138 mM NaCl, 2.7 mM KCl, and 0.05% Tween 20. The antigen–antibodies complexes were detected by Clarity Western ECL Substrate 1,705,061 (Bio-Rad) using horseradish peroxidase-conjugated goat anti-mouse IgG or anti-rabbit IgG as secondary antibody. Digital images were acquired using ChemiDoc Touch Imaging Systems (Bio-Rad), and band density was measured by Bio-Rad software (Bio-Rad, Image Lab 6.0).

### Histology, PAS staining, goblet cell density measurements, and quantification of focus score

Eyes and ocular adnexa were excised, fixed in 10% formalin, paraffin-embedded, and cut into 5-µm sections using a microtome (Microm HM 340E, Thermo Fisher Scientific). Sections were stained with Periodic Acid–Schiff (PAS) reagent. The goblet cell density was measured in the superior and inferior bulbar and tarsal conjunctiva using NIS-Elements software (AR, version 5.20.2; Nikon Melville, New York) and expressed as the number of positive cells per millimeter [35].

Lymphocytic infiltration foci were counted in hematoxylin and eosin-stained (H&E) sections of LGs by standard light microscopy using a 10X objective (Nikon, Eclipse E400) by two observers. A minimum of 50 mononuclear cells was counted as one focus, and the total number of foci per gland was recorded. Slides were scanned to obtain digital images using PathScan Enabler V (Meyer Instruments, Houston, Texas) and were calibrated according to the manufacturer’s instructions (2.54 µm/px) using NIS Elements software. The LGs’ total area was measured using the “autodetect area” function of the Nikon Elements software or was manually circumscribed using the polyline function. Finally, focus scores were calculated by dividing the number of foci per mm^2^ and quantifying the number of inflammatory cell foci per 4 mm^2^ tissue area.

### Immunohistochemistry and immunofluorescence of LG

Left exorbital LGs were excised, embedded in OCT compound, and flash frozen in liquid nitrogen. Sagittal 10-µm sections were cut with a cryostat (HM 500; Micron, Waldorf, Germany) and placed on glass slides stored at −80 °C.

Immunohistochemistry was performed to detect CD4, CD19, and MHC II cells in the LG using rat anti-CD4 (clone H129.19, BD Biosciences/BD Pharmingen, San Diego, California), rat anti-CD19 (clone 6D5, Fisher Scientific), and rat-anti-MHC II (I-A/I-E,clone M5/114.15.2, BD Pharmingen) antibodies and appropriate biotinylated secondary antibodies (BD Biosciences) and a VECTASTAIN Elite ABC kit using NovaRED reagents (Vector Laboratories, Burlingame, California) as previously described [35, 36]. Images were acquired and photographed with a microscope equipped with a digital camera (Eclipse E400 with a DS-Fi1; Nikon). Negative controls were sections where the primary antibody was omitted.

Immunofluorescence was performed to detect CD68 using an anti-rat CD68 antibody (clone FA-11, Bio-Rad) using cold acetone fixation and 20% goat serum as a blocking solution. An AlexaFluor-488 goat-anti-rat secondary antibody was used (Jackson Immune Research, West Grove, Pennslyvania), and propidium iodide (Sigma-Aldrich) was used as nuclear counterstaining. Digital confocal images were captured with a laser scanning confocal microscope (Nikon A1 RMP, Nikon) wavelength of 400–750 nm. The images were processed using the NIS Elements 4.20 version. Negative controls were sections where the primary antibody was omitted.

### Measurement of corneal barrier function

Corneal barrier function was assessed by quantifying corneal epithelial permeability to 70-kDa Alexa Fluor® Oregon Green Dextran (OGD; Thermo Fisher), according to a previously published protocol [37]. Briefly, 1 µL of a 50 mg/mL OGD solution was applied to the ocular surface 1 min before euthanasia, which was performed using excess isoflurane followed by cervical dislocation. Corneas were rinsed with 2 mL of PBS and photographed with a stereoscopic zoom microscope (model SMZ 1500; Nikon) under fluorescence excitation at 470 nm. OGD staining intensity was quantified in digital images by measuring the mean fluorescence intensity within a 2-mm diameter circle placed on the central cornea using NIS Elements software (version AR, 5.20.02). This assessment was conducted independently by two observers. The mean intensity of the right and left eyes was averaged, and then the resulting mean from biological replicates was calculated and analyzed.

### Statistical analysis

Based on pilot studies, the sample size was calculated with StatMate2 Software (GraphPad Software, San Diego, California). Statistical analyses were performed with Graph Pad Prism software (GraphPad Software, version 9.2). Data were first evaluated for normality with the Kolmogorov–Smirnov normality test. Appropriate parametric (t-test) or non-parametric (Mann–Whitney) statistical tests were used to compare the two age groups. Whenever adequate, one-way or two-way ANOVA or Kruskal–Wallis followed by post hoc tests were used. The final sample per experiment is shown in the legends**.**

## Results

### Aging is associated with the production of proinflammatory factors

**Fig. 1:**
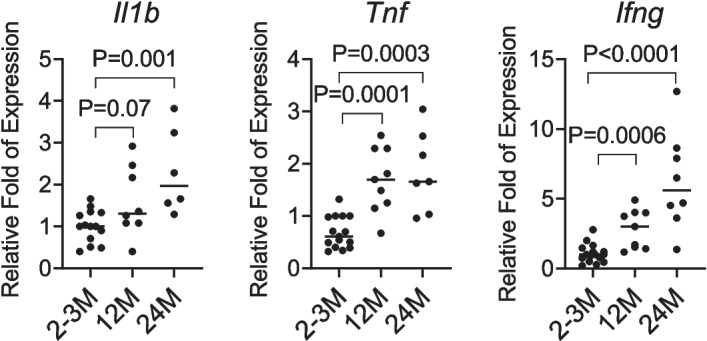
Aging increases inflammatory cytokines in the LG. LGs of female C57BL/6J mice of different ages were collected, RNA was extracted, and qPCR was performed. Relative fold changes of interleukin-1-β (*Il1b*), TNF (*Tnf*), and interferon-γ (*Ifng*) in LGs of 2-3M, 12M and 24M mice. Each dot represents one mouse. P value as shown. One-way ANOVA with Dunn’s multi-comparison test. 2-3M, *n* = 10; 12M, *n* = 8; 24M, *n* = 8

As organisms age, a heightened inflammatory response is characterized by elevated expression of inflammatory cytokines. IL-1β and TNF are the central mediators of inflammation and are involved in various age-related diseases [15, 38, 39]. IFN-γ, primarily produced by NK and T cells, plays a crucial role in immune responses and is upregulated with aging [40, 41]. The elevated mRNA levels of *Il1b*, *Tnf*, and *Ifng* mRNA generally indicate an enhanced inflammatory state that contributes to the development and progression of age-related pathologies. To investigate changes in the inflammatory milieu, we performed qPCR on LGs from mice aged 2–3 M, 12 M, and 24 M. Our results demonstrate a progressive increase in *Il1b*, *Tnf*, and *Ifng* mRNA levels with age (Fig. 1), indicating that aging induces an inflammatory response in the LG.

### CR supports lacrimal gland function in middle-aged mice

**Fig. 2:**
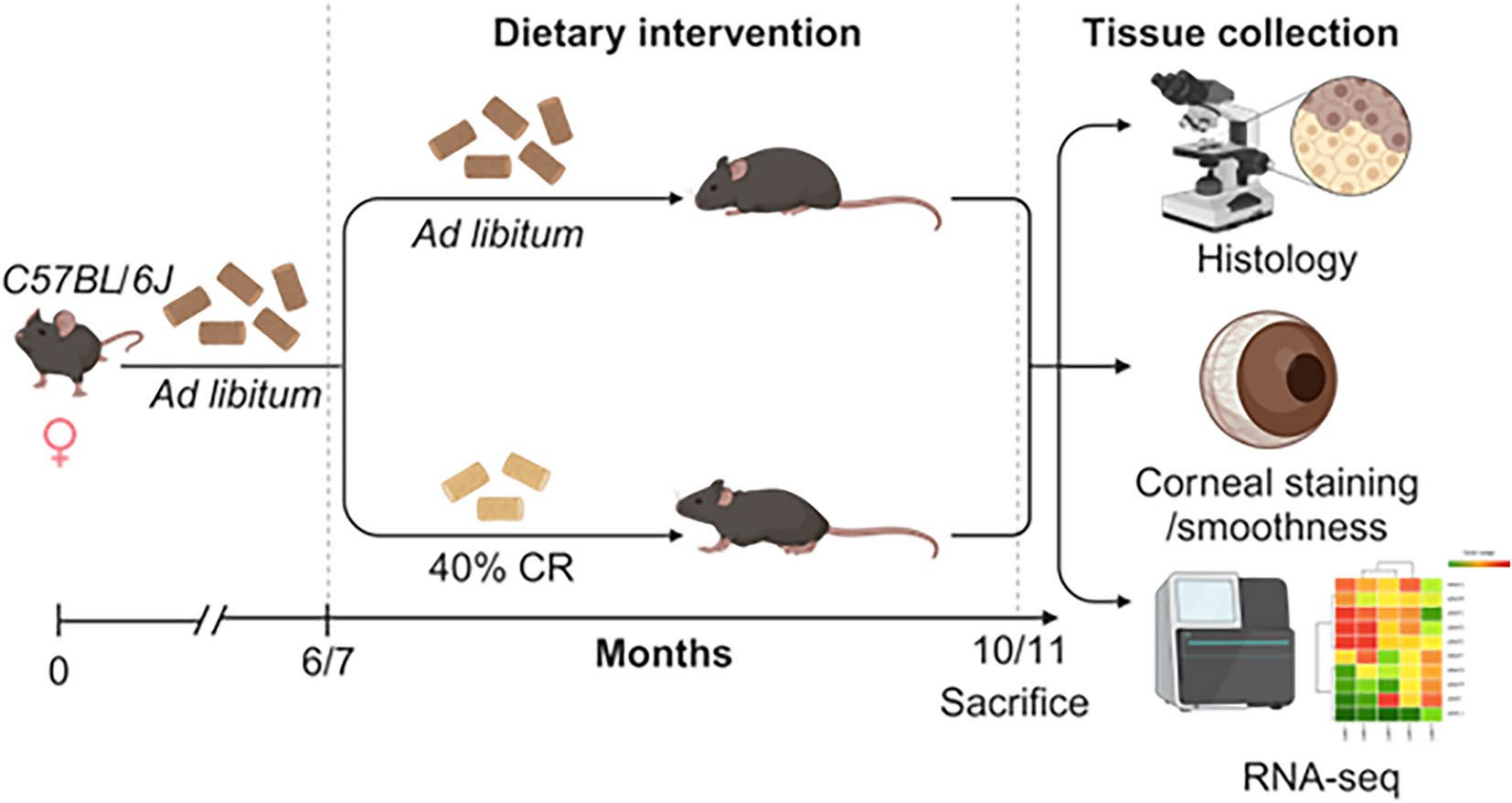
Experimental design. Female C57BL/6J mice were subjected to AL or early 40% CR from 7M to 11M. LGs were excised for histology and gene expression using qPCR and bulk RNA sequencing. RNA samples from AL (LG, *n* = 4; cornea, *n* = 5; conjunctiva *n* = 4) and CR (LG, *n* = 4; cornea, *n* = 5; conjunctiva, *n* = 5) mice were sequenced. Significant DEGs (FC > 1.5 or < -1.5, FDR < 0.05) lists were uploaded to Metascape to identify the biological pathways significantly modified in CR. Corneal barrier function was measured as the uptake of fluorescent dye. Eyes were excised for histology and used to measure goblet cell density

**Fig. 3:**
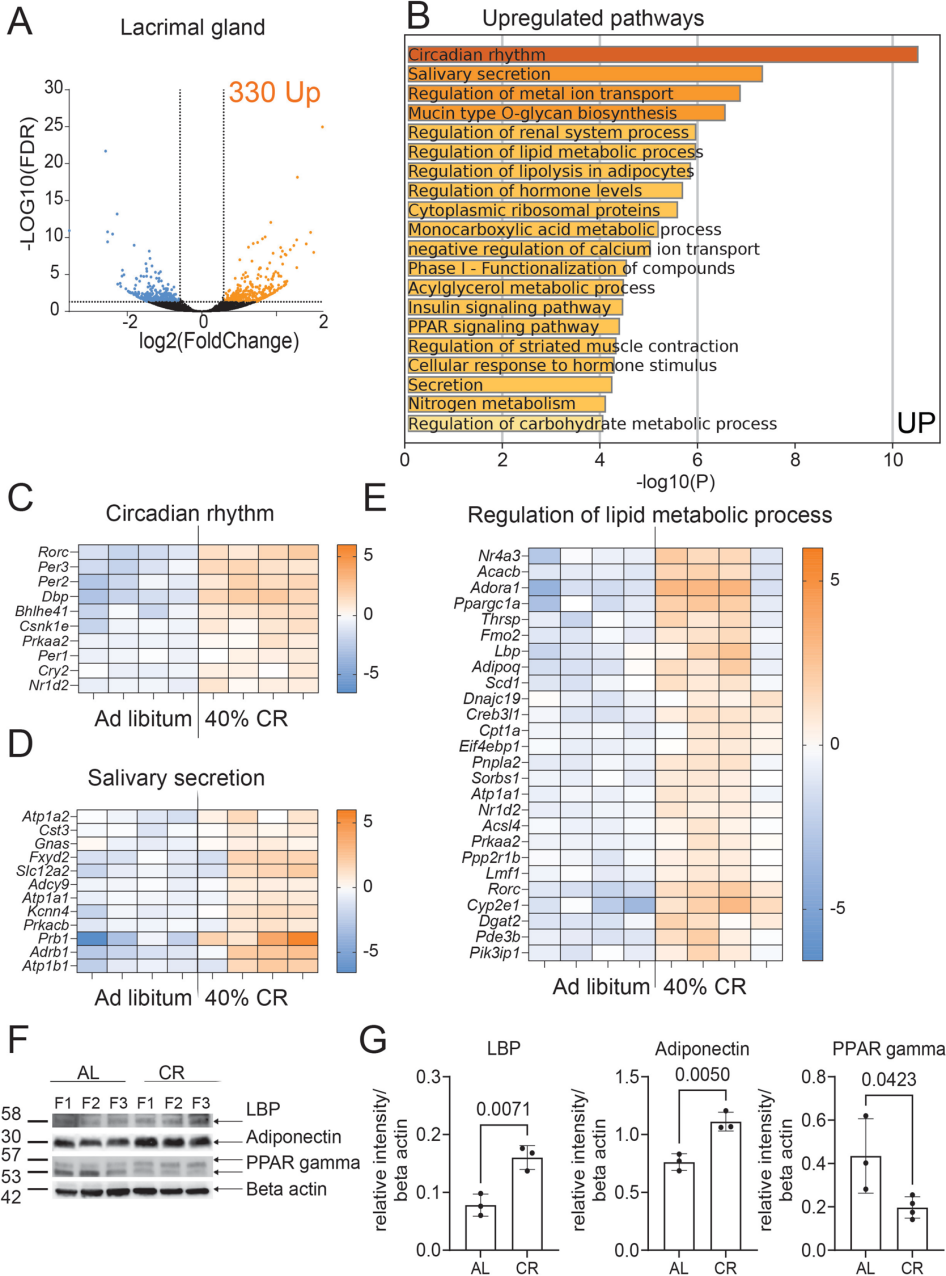
Transcriptomic reprogramming induced by preventive CR in LGs. **A** Volcano plot of DEGs between CR (*n* = 4) and AL (*n* = 4) mice. 330 significant (*p*-adj < 0.05) DEGs that are (**B**) upregulated (FC > 1.5, orange) were submitted for pathway analysis using Metascape. The top 10 significant enriched pathways are shown. Heatmap of significant DEGs involved in Circadian rhythm (**C**), Regulation of lipid metabolic process (**D**), and Salivary Secretion (**E**)  pathways. **F** Western blot digital images of LG lysates probed with anti-LPB, adiponectin and PPAR-gamma antibodies. Each lane is a different biological LG. F = female. **G** Densitometry quantification of data shown in F. Bands were quantified and the ratio to beta-actin is shown. Each dot represents one LG. The Mann-Whitney *U*-test was used. P value as shown

**Fig. 4:**
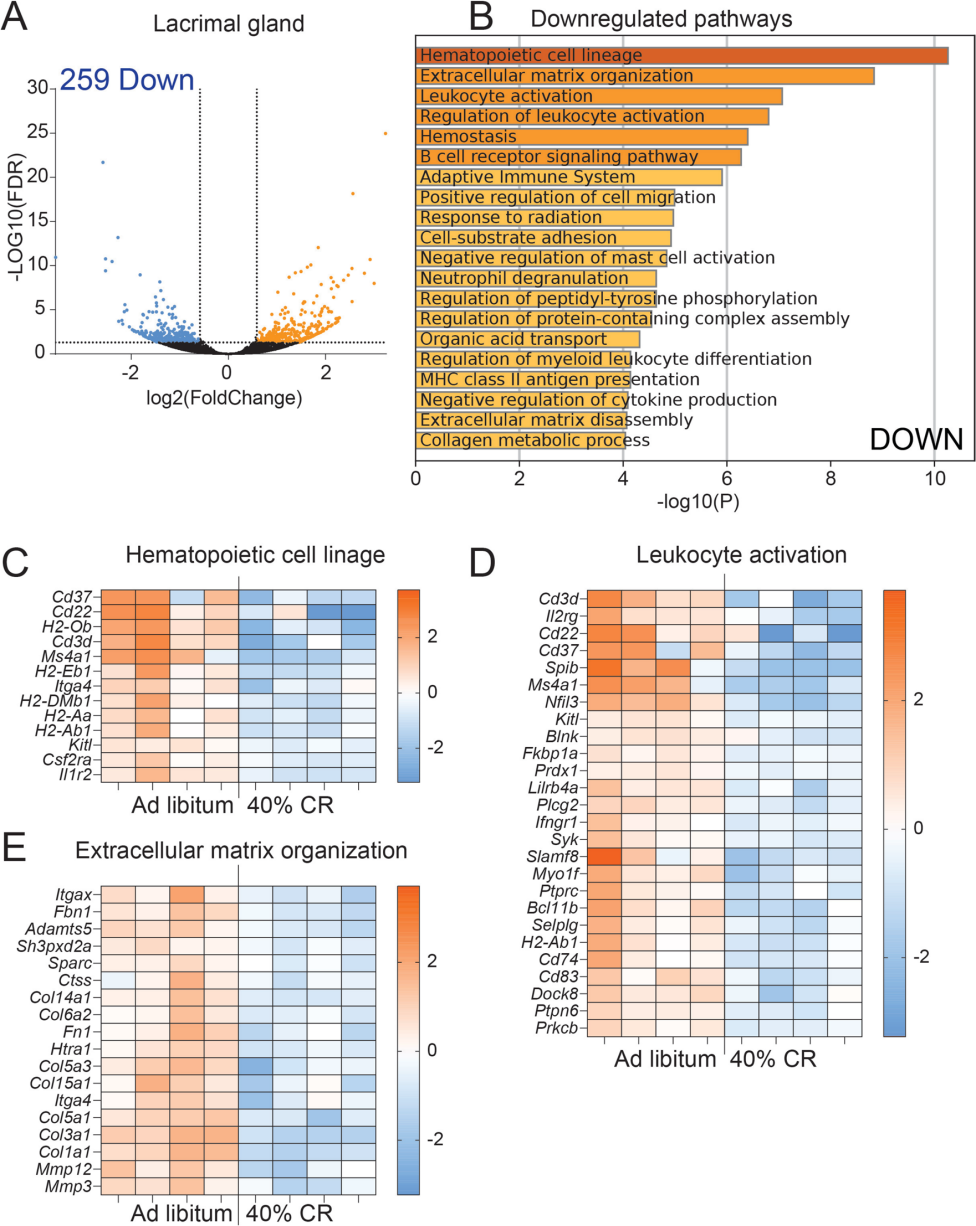
Pathways downregulated by preventive CR in LGs. **A** Volcano plot of DEGs between CR (*n* = 4) and AL (*n* = 4) mice. 259 significant (*p*-adj < 0.05) DEGs that are (**B**) downregulated (FC < 1.5, blue) were submitted for pathway analysis using Metascape. The top 10 significant enriched pathways are shown. Heatmap of significant DEGs involved in Hematopoietic cell linage (**C**), Leukocyte activation (**D**) and Extracellular matrix organization (**E**) pathways

To assess whether CR can slow down inflammation in the LG of adult mice, 6–7 M mice were assigned to either an AL diet or a 40% CR for 4 months (Fig. 2). Mice were weighed weekly during the 16 weeks of CR (Supplemental Fig. 1). At euthanasia, exorbital LGs were collected for bulk RNA sequencing or immunostaining. To identify genes affected by CR in the LG, we compared the LG transcriptomes of mice on a CR diet (*n* = 4) with those on an AL diet (*n* = 4) using bulk RNA sequencing (Figs. 3 and 4). Differentially expressed genes (DEGs) were identified based on a fold change (FC) of ≥  −1.5 or ≤ 1.5 and an adjusted *p*-value of < 0.05. This analysis revealed 589 DEGs: 330 upregulated and 259 downregulated (Figs. 3A, B and 4A, B, Supplementary File 1). The sets of upregulated and downregulated genes were further analyzed using Metascape to identify major biological pathways significantly enriched in CR LGs (Supplementary Files 2 and 3).

The most significantly upregulated ontology term identified was “circadian rhythm” (Fig. 3A–C). Notably among the DEGs, we identified the *Per* and *Cry* genes, which are key components of the circadian core oscillator complex [42, 43]. We also observed that *Bhlhe41* and *Dbp* are upregulated*. Bhlhe41* and *Dbp* are transcriptional regulators of circadian rhythms [44, 45]. Aging has been reported to disrupt the molecular mechanisms regulating the circadian clock in many tissues, including the LG [46]. However, it has also been reported that CR mice consume their entire daily food within approximately 1 h, whereas AL-fed mice maintain a more constant energy intake throughout the day [47]. Therefore, it is not surprising that CR enhances the expression of circadian clock regulators.

The second most significantly upregulated pathway, “Salivary secretion,” was associated with genes that have been involved with the secretory function of the LG (Fig. 3B, D). This pathway included numerous ion channels (*Kcnn4*, *Cftr*,* Scnn1a*,* Scnn1b*), transporters (*Atp1a1*, *Atp1b1*,* Atp1a2*, *Slc12a8*, *Slc12a2*), ribosomal proteins, and glycosylation-related enzymes (*Galnt3*,* Galnt15*, *Gcnt1*). qRT-PCR analysis of additional LG samples confirmed the increased expression of *Slc12a2*, *Kcnn4*, and *Prb1*, while *Fxyd2* had a non-significant increase (Supplemental Fig. 2A). The upregulation of protein translation and maturation, along with enhanced ion secretion and exchange pathways under CR, suggests increased protein synthesis and secretory activities. Other pathways enriched in CR-upregulated DEGs were related to lipid metabolism as shown by the predicted pathways “regulation of lipid metabolic process” and “regulation of lipolysis in adipocytes” (Fig. 3E); DEGs within these pathways were involved in mitochondrial β-oxidation (*Cpt1a*) and fatty acid biosynthesis, which corresponds to the creation of fatty acids from acetyl-CoA and occurs in several organelles. Additionally, DEGs from these pathways were associated with the upregulation of key regulators of mitochondrial bioenergetics and lipid metabolism, including *Ppargc1a*, *Adipoq*, *Adora1*, *Cyp2e1*, and *Prkaa2*. qRT-PCR analysis of additional samples confirmed the increased expression of *Adipoq*, *Adora1*, *Cyp2e1*, and *Lbp* in CR LGs compared to AL LGs (Supplemental Fig. 2B). The increased expression of lipopolysaccharide-binding protein (LBP, encoded by *Lbp*), and adiponectin, which regulates glucose and fatty acid oxidation, was validated using western blotting (Fig. 3F, G). Consistent with an increase in fatty acid oxidation for energy production rather than lipogenesis for fat storage, CR decreased peroxisome proliferator-activated receptor gamma (PPAR-γ) protein levels, in agreement with the literature [48, 49].

In summary, our data suggest that CR may support LG function by enhancing secretion, mitochondrial activity, and lipid metabolic processes for increased energy production and expenditure. These processes have previously been found to be impaired by chronic inflammation in the LGs and salivary glands with aging [50, 51].

### CR maintains LG function by delaying the progression of inflammation and modulating the ECM

The 259 genes significantly downregulated by CR (Fig. 4A–E) were notably enriched in several immune-related pathways (Supplementary files). Among the top 10 most enriched pathways were “Hematopoietic cell lineage,” “Extracellular matrix organization,” “Leukocyte activation,” “Hemostasis,” “B cell receptor signaling pathway,” “Adaptive immune system,” and “others” (Fig. 4B). A heatmap of the DEGs involved in hematopoietic cell linage is shown in Fig. 4C. We observed significant downregulation of genes expressed by T cells (*Cd3d*), B/plasma cells (*Cd22*, *Cd37*, *Jchain*), MHC-II molecules (*H2-Aa*, *H2-Eb1*, *H2-DMb1*, *H2-Ob*), and genes involved in hematopoietic progenitor cell proliferation and differentiation (*Ptpn6*, *Kitl*) (Fig. 4C). The downregulation of the pan-leukocyte marker *Ptprc* (encoding CD45) and *Cd83* (expressed by activated immune cells), as well as markers of the myeloid (*Cd74*) and lymphoid (*Ms4a1)* lineages, collectively suggests a reduced presence of these cells in CR LGs compared to AL-fed mice (Fig. 4D).

The second most significant pathway downregulated by CR was “Extracellular matrix organization” (Fig. 4E). This pathway notably included genes encoding extracellular matrix (ECM) components, such as *Fn1*, *Fbn1*, and various collagen genes and metalloproteinases like *Mmp3*, *Mmp12*, *Adamts5*,* Adamts15*, and *Ctss*. We previously reported that cathepsin S (encoded by *Ctss*) has a pathogenic role in aged mice [37, 52]. These findings suggest reduced ECM remodeling in CR-treated LGs. ECM remodeling and epithelial-mesenchymal transition (EMT) are early indicators of LG inflammation [14, 53].

Our data suggest CR may enhance LG function by preventing age-associated changes in immune response and ECM remodeling.

### CR decreases immune infiltration in the lacrimal gland

**Fig. 5:**
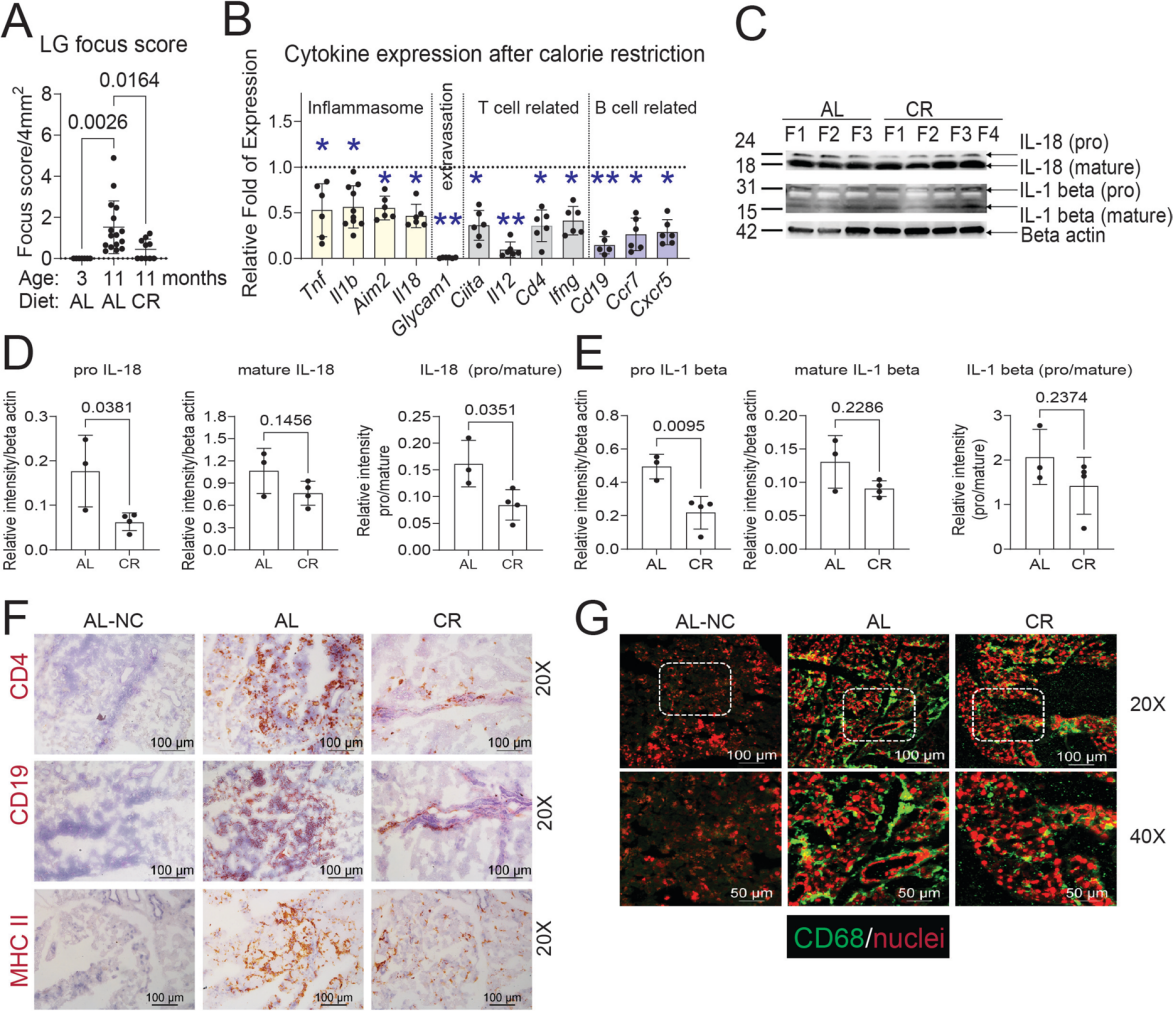
CR changes inflammatory markers in the LG. **A** Focus score of LG infiltrates in 3M AL (*n* = 7), 11M AL (*n* = 16) and 11M CR (*n* = 10). **B **Relative fold expression of transcripts related to inflammasome, extravasation of lymphocytes, and T and B cell-related genes. The dotted line indicates the AL level, and the individual bar graphs show CR levels. Each dot represents one animal. * *P* < 0.05; ** *P* < 0.01. *n* = 5-6/group but *Il1b* (*n* = 10). **C** Western blot digital images of LG lysates probed with anti-IL-18 and IL-1 beta antibodies. Each lane is a different biological LG. F = female. (D-E) Densitometry quantification of IL-18 (**D**) and IL-1β (**E**).  Bands were quantified and the ratio to beta-actin is shown. Each dot represents one LG. The Mann-Whitney *U*-test was used. *P* value as shown. **F** Representative images of LG sections stained with anti-CD4, anti-CD19, and anti-MHC II (EA/IE). Positive reactions are identified in red. Scale bar 100µm. **G** Representative merged images of laser confocal microscopy of frozen sections stained with anti-CD68 (green) and propidium iodide (red) nuclear counterstaining. The top rows show images taken at 20X (scale bar = 100µm), and the bottom rows show magnified images of the dotted squares with a scale bar of 50µm

A key feature of aging observed in middle-aged animals is the onset of immune infiltration in various organs, including the LG. We have previously demonstrated that the LG develops mononuclear infiltrates as quantified by the focus score, which measures immune infiltrates (> 50 mononuclear cells) that organize themselves into immune foci [15]. While immune cells were still present around the ducts in the CR LGs, they did not form large foci as typically seen in aging, and the focus scores were significantly lower than in the AL group (Fig. 5A).

To validate this decrease in infiltrating immune cells, we performed individual qRT-PCRs on the other LG from the same animals (Fig. 5B). We selected genes previously shown to be upregulated with aging (Fig. 1 and [15, 39, 54]). CR significantly decreased the expression of genes involved in inflammasome signaling, such as *Tnf*, *Il1b*, *Aim2*, and *Il18* (50–60% reduction), as well as genes related to leukocyte extravasation (*Glycam1*, 95% reduction), Th1 priming [*Ciita* (MHC II, − 64%), *Il12* (− 81%), *Cd4* (− 65%), *Ifng* (− 60%)], and B cell function [*Cd19* (− 86%), *Ccr7* (− 74%), and *Cxcr5* (− 61%)] compared with the AL group (dotted line, Fig. 5B).

We further validated the IL-18 and IL-1β protein expression levels, since these proteins are critical markers involved in the inflammasome pathway using western blot (Fig. 5C). Consistent with mRNA levels, CR significantly decreased the abundance of pro-IL-18 and pro-IL-1β (Fig. 5D and E), suggesting that CR reduces the pro-inflammatory signals promoting their transcription. These results indicate that CR has profound effects on inflammatory pathways within the LG.

Immunohistochemistry further validated these findings at the protein level (Fig. 5F). CR-treated LGs had decreased protein expression of immune cell markers CD4, CD19, and MHC II (encoded by *Citta* gene) (Fig. 5F). By immunofluorescence, we also observed a qualitative reduction in the number of CD68^+^ cells, a protein marker present in macrophages, monocytes, and myeloid dendritic cells (Fig. 5G).

These results indicate that CR reduces the aging-associated immune response within the LG, reshaping the immune landscape.

### CR delays the onset of dry eye development

**Fig. 6:**
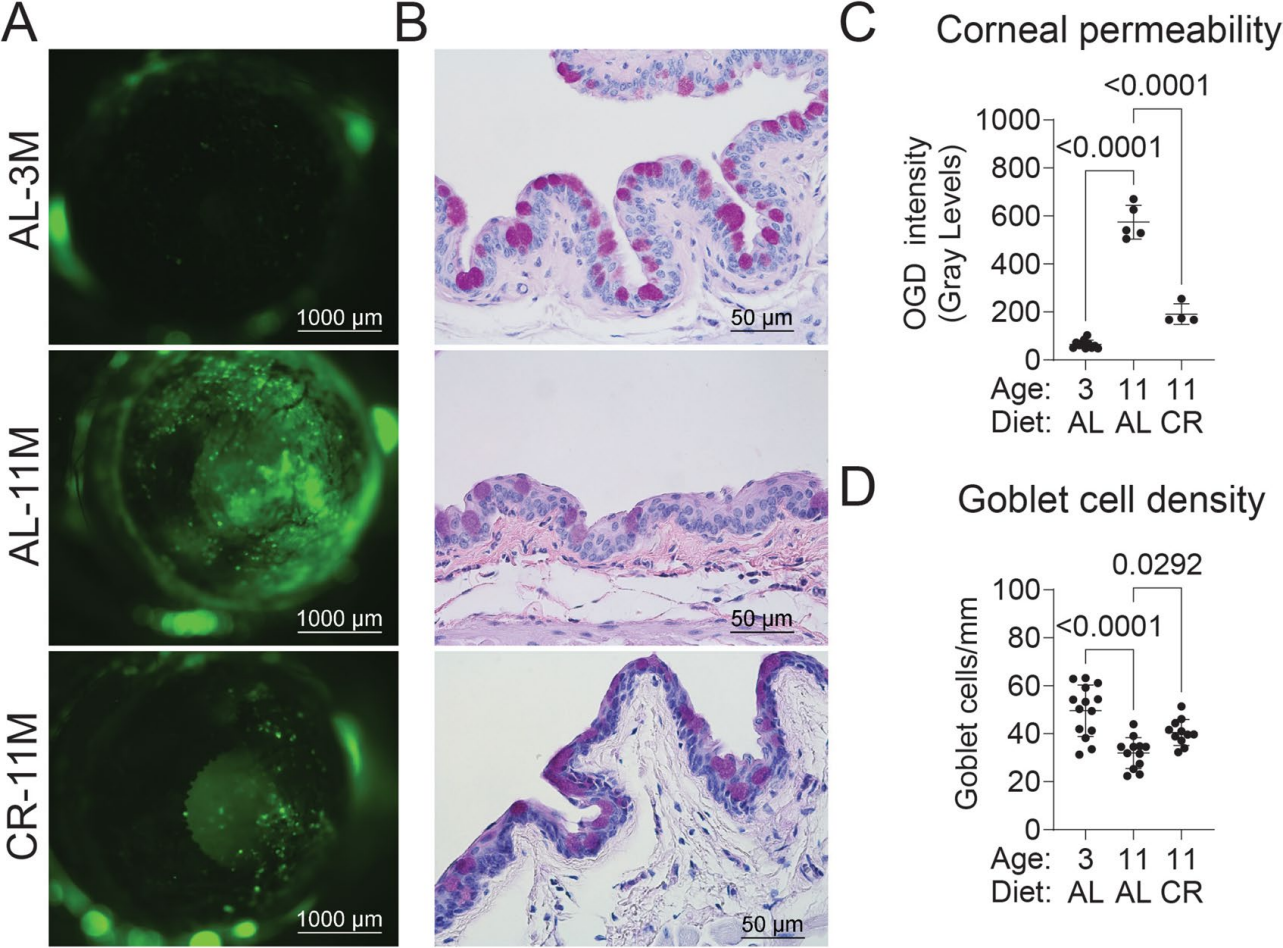
CR in mice for 4 months prevents the development of dry eye in mice. Representative images (**A**) and cumulative data (**C**) of corneas stained with Oregon Green Dextran-488 (OGD) in 11M CR mice. Greater uptake of the dye indicates corneal barrier disruption. Representative images (**B**) and cumulative data (**D**) of PAS-stained paraffin sections identifying goblet cells (purple-magenta). Cumulative data on the graph; each dot represents one animal (average of right and left eyes). Mann-Whitney *U*-test was used. *P* value as shown

Aged mice develop dry eye, as characterized by corneal barrier disruption and conjunctival goblet cell loss. These hallmarks of dry eye disease [7] are seen as early as 10–11 M [27, 37, 55]. We evaluated whether CR protects the ocular surface from developing dry eye by analyzing corneal barrier function with fluorescent dye uptake [56] and by measuring conjunctival goblet cell density in paraffin-embedded eye sections. Young mice (3 M) receiving food AL were included as additional controls. Eleven-month AL-fed mice exhibited typical dry eye signs, including increased corneal barrier disruption (Fig. 6A, C) and conjunctival goblet cell loss (Fig. 6B, D). In contrast, mice subjected to CR demonstrated significantly improved corneal barrier integrity and increased conjunctival goblet cell density. These findings indicate that CR prevents the development of age-related dry eye.

**Fig. 7:**
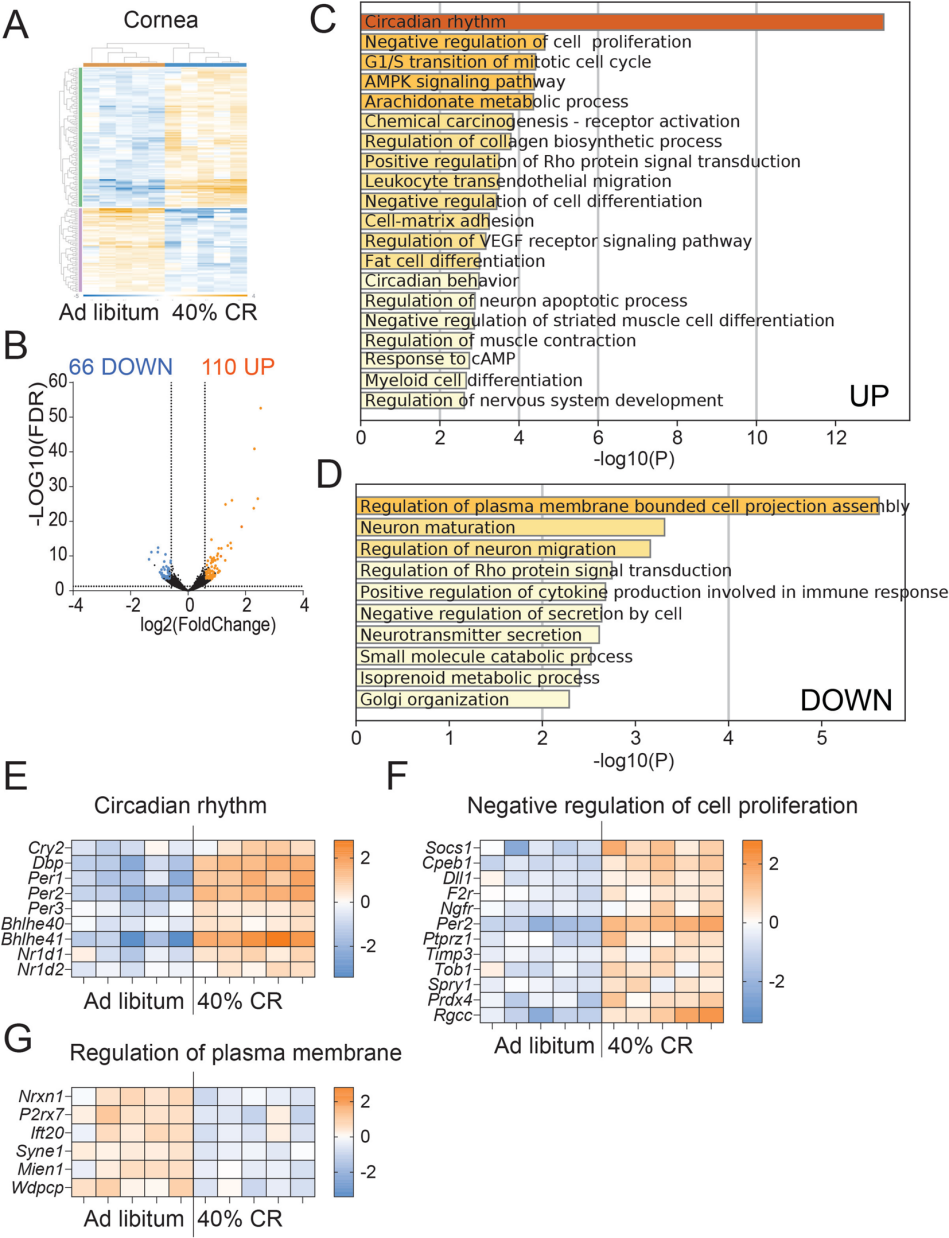
CR in mice for 4 months alters the cornea transcriptome. Corneal epithelium was collected by scraping and total RNA was isolated. Bulk RNA sequencing was performed as described in the methods. **A** Heatmap showing overall changes after CR. **B** Volcano plot of DEGs between CR (*n* = 5) and AL (*n* = 5) mice. 176 significant (p-adj < 0.05, FC > 1.5, orange; FC < 1.5, blue) DEGs were submitted for pathway analysis using Metascape. **C**-**D** The top significant enriched pathways are shown after uploading upregulated (**C**) or downregulated (**D**) DEGs. **E**-**G** Heatmaps of significant DEGs involved in Circadian rhythm (**E**), Negative regulation of cell proliferation (**F**) and Regulation of plasma membrane (**G**)

**Fig. 8:**
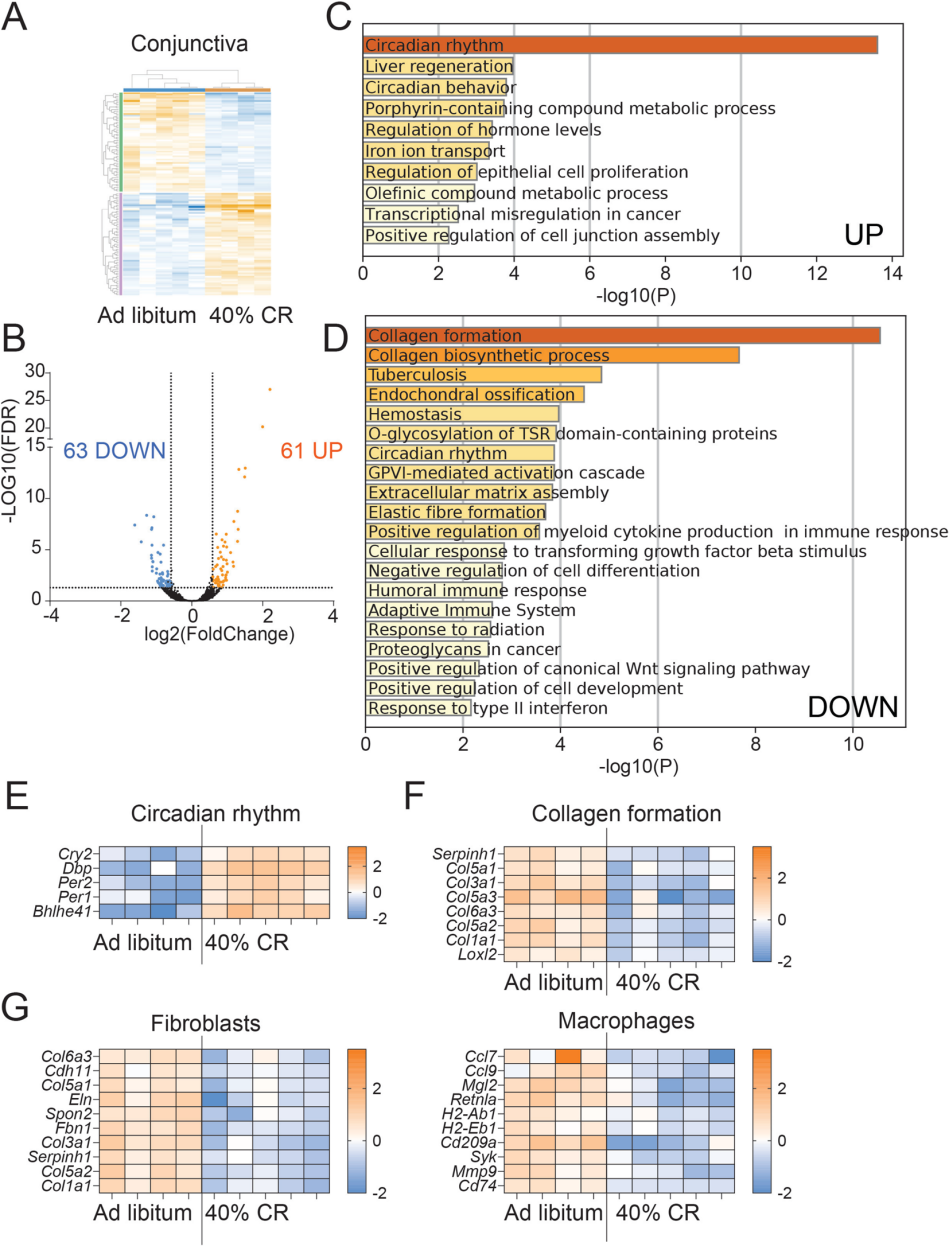
CR in mice for 4 months alters the conjunctival transcriptome. Conjunctiva was excised and the total RNA was isolated. Bulk RNA sequencing was performed as described in methods. **A** Heatmap showing overall changes after CR. **B** Volcano plot of DEGs between CR (*n* = 4) and AL (*n* = 5) mice. 124 significant (p-adj < 0.05, FC > 1.5, orange; FC < 1.5, blue) DEGs were submitted for pathway analysis using Metascape. **C**-**D** The top significant enriched pathways are shown after uploading upregulated (**C**) or downregulated (**D**) DEGs. **E**-**G** Heatmaps of significant DEGs involved in Circadian rhythm (**E**), Collagen formation (**F**) and predicted cell types (**G**)

The pathways that CR modulates in the cornea and conjunctiva were investigated using bulk RNA sequencing (Figs. 7 and 8, Supplemental Files 5 and 6). In the corneal epithelium, we observed that CR differentially modulated 176 genes (Fig. 7 A, B). Metascape analysis identified that similarly to the LG, the “circadian rhythm” was the top most upregulated predicted pathway (Fig. 7C) while the top most downregulated pathways were the “Regulation of plasma membrane bounded cell projection assembly” (Fig. 7D). Upregulation of *Per1*, *Nr1d1*, *and Nr1d2* genes involved in the circadian rhythm was observed in CR group (Fig. 7E). The second mostly upregulated pathway was “Negative regulation of cell proliferation,” suggesting that CR decreased epithelial turnover and cellular desquamation. The DEGs related to “regulation of plasma membrane” are shown in Fig. 7G.

In the conjunctiva, we observed that 124 genes were differentially modulated by CR (Fig. 8A, B). Metascape analysis indicated that similar to the LG and cornea, the “Circadian rhythm” was the topmost upregulated predicted pathway (Fig. 8C) while the topmost downregulated pathway was “Collagen formation” (Fig. 8D). Upregulation of *Cry2*, *Dbp*, *Per2*, *Per1*, and *Bhlhe41* was observed in the CR group (Fig. 8E). The DEGs related to “Collagen formation” are shown in Fig. 8F and include *Serpinh1*, several collagen subunits, and *Loxl2*. *Serpinh1* encodes HSP47, a serine proteinase inhibitor. HSP47 expression is increased in conjunctival fibrotic diseases, including ocular cicatricial pemphigoid and post-glaucoma surgery [57, 58]. ROSALIND analysis using the PanglaoDB database [59] identified fibroblasts (*P* = 0.003) and macrophages (*P* = 0.004) as the top cell types modulated by CR. The genes related to these cell types are shown in Fig. 8G.

In summary, our results demonstrate that CR, initiated at the beginning of middle age, helps to maintain a healthier ocular surface and LGs by preserving mitochondrial, secretory activity, and LG metabolism while reducing inflammatory changes and ECM remodeling. These changes are also present in the cornea and conjunctiva. Collectively, these effects contribute to improving the age-related dry eye phenotype.

## Discussion

This study investigated the effects of calorie restriction (CR) on improving age-related dry eye. First, we established that aged LGs have increased expression of the inflammatory transcripts *Il1b*,* Tnf*, and *Ifng*. We then analyzed LGs from mice with either AL or CR access to food. We demonstrated that 4-month-long CR in adult mice reduces inflammation in the LG and on the ocular surface and affects genes related to ECM, the immune response, LG secretion, and circadian rhythms. We also identified similar changes in the cornea and conjunctiva.

The most significant upregulated predicted biological pathway in the LG, cornea, and conjunctiva was related to circadian rhythm. These observed circadian rhythm changes were likely driven by the distinct eating patterns of CR mice, as they consumed their daily food within a more restricted time compared to AL fed mice. Given that the timing of food intake can influence the physiological effects of CR, further studies are needed to dissect the specific contribution of intermittent fasting versus reduced calorie intake to the observed CR-mediated changes in the LG. Such research could help elucidate the mechanistic underpinnings of CR’s impact on the LG and its potential therapeutic implications for age-related ocular conditions.

Of the other upregulated biological pathways, CR significantly improved genes related to LG secretion, suggesting a functional enhancement of glandular cells. This finding aligns with a previous study by Kawashima and coauthors [22], which reported a 35% increase in tear volume and acinar cell density in CR-treated rats, as assessed by tear volume tests and histological examination. Other pathways upregulated by CR in the LG were enriched in genes associated with lipid metabolism and fatty acid oxidation. Using western blots, we confirmed an increase in LBP and adiponectin protein levels and a decrease in PPAR-γ. These results agree with the literature and show a global effect of CR on the lipid metabolism in the LG [48, 49]. These pathways observed in CR-treated mice may play a dual role. First, fatty acid oxidation can provide energy to acinar cells, supporting their secretory function. Second, lipid metabolic processes have anti-inflammatory properties [50], potentially reducing immune cell activation and the production of pro-inflammatory lipid mediators. In our previous study using the NOD.H2^b^ mouse model for Sjögren’s disease [60], we reported that chronic inflammation is associated with the downregulation of genes involved in fatty acid oxidation, the TCA cycle, and de novo lipid biosynthesis, correlating with disease progression, while cholesterol synthesis and accumulation were increased. Furthermore, a comparison of aged 24 M LGs to NOD.H2^b^ LGs showed that aging shares many of the same altered pathways with the NOD.H2^b^ mice [54]. This suggests that disruption in fatty acid oxidation and lipid biosynthesis pathways could represent a common mechanism of chronic inflammation leading to LG dysfunction, one that CR has the potential to reverse.

The most significantly downregulated pathways in CR-treated mice in the LG and conjunctiva were pathways associated with inflammation, immune cells, and ECM. For instance, the downregulation of the “hematopoietic cell lineage” and “leukocyte activation” pathways suggest that CR reduces the production and activation of lymphoid origin cells [61]. Chronic inflammation associated with aging is often driven by excessive leukocyte recruitment and activation. Additionally, the observed decrease in the “regulation of leukocyte cell–cell adhesion” pathway suggests a reduction in the processes that mediate leukocyte attachment to endothelial cells or other surfaces, such as ducts or acini. This mechanism is critical during inflammation, as it is a key step in recruiting leukocytes to inflamed or injured tissues. Likewise, our qPCR results identified an 85% reduction in *Glycam1*, one of the peripheral lymph node addressins [62] expressed in the aged murine LG [54]. By decreasing leukocyte adhesion, CR may shift the immune environment towards a less inflammatory state, consistent with findings from models of chronic inflammatory diseases. Oxidative stress and inflammation are closely interconnected, often amplifying each other, and both have been widely implicated in the aging process [15, 63–65]. Oxidative damage [15] and inflammation have been associated with aging and have thus been suggested as potential targets for combating age-related diseases [66]. It has been reported that CR decreases oxidative stress markers and cytokines IL-6 and TNF levels in the LG compared to an age-matched group fed AL [23]. Likewise, we have reported that anti-TNF eye drops have a beneficial effect on goblet cell density and LG immune infiltration [39], suggesting that controlling inflammaging is a key component for aging-related interventions.

The second major biological pathway downregulated by CR was the synthesis and remodeling of ECM. CR decreased gene expression in collagen production and other ECM-related proteins, including *Mmp3*,* Mmp12*, and *Ctss*. This downregulation likely limits ECM deposition, preventing tissue stiffening and preserving elasticity, both critical for maintaining organ function during aging. We published that *Ctss*^−/−^ mice are resistant to age-related dry eye [37]. Furthermore, dry eye disease is associated with increased LG gland tissue stiffness, which has been suggested as a diagnostic of dry eye [67]. ECM organization and remodeling are also key features of inflammation [68, 69], and downregulation of these pathways was also observed in the conjunctiva. The main cell types modulated by CR in the conjunctiva were fibroblasts and macrophages. These cells can sense environmental changes through ECM [68]. These findings align with reports from other tissues showing that CR enhances and reduces markers of aging [28].

CR likewise improved the overall health of the LG. We have previously described how aging is accompanied by the development of ectopic lymphoid structures in the LG [54]. This age-associated increase in infiltrating immune cells in the LG is difficult to reverse. Excitingly, we described here how 40% CR was able to significantly reduce the number and size of infiltrating foci in the LGs. This suggests an improvement in the age-related inflammation that occurs with aging. We confirmed the reduction in mononuclear infiltrates with immunohistochemistry and immunofluorescent staining for immune cell markers CD4, CD19, MHC II, and CD68. Both identified a reduction in immune cell proteins in the CR mice, confirming that the reduction of foci in the histological LG sections resulted in a lower presence of immune cells in the LGs.

In addition to improving the LG, we saw an improvement of aging-related dry eye signs on the ocular surface as well. Hallmarks of dry eye include aging-related goblet cell density loss and corneal barrier disruption [55]. CR protected both goblet cell density and the corneal barrier. It is possible that the protection of the corneal barrier resulted from a healthier tear film due to the increased presence of goblet cells and the increased health of the LG. However, we also observed significant transcriptional changes in the cornea after CR. We recently demonstrated that aging alters the corneal epithelium transcriptome [70], but the earliest age was 12 months. Our present study shows that CR in young mice can profoundly alter the corneal transcriptome and suggests that these early changes in cell proliferation, neuron maturation, and other pathways are related to aging. Taken together, our findings suggest that CR had a holistic effect on the eye and improved multiple tissues associated with aging-related dry eye.

While CR alone has promising therapeutic potential, another promising avenue of treatment for age-related inflammation is combining CR with other interventions to alleviate age-related exocrine gland dysfunction. For example, pharmacological agents targeting mitochondrial metabolism, inflammation, oxidative stress, or ECM remodeling may provide similar benefits. Thus, combining CR with antioxidant supplementation or anti-inflammatory drugs may yield synergistic effects. Future research should investigate these combinations to determine whether they offer enhanced protection against age- and inflammation-related gland dysfunction.

Overall, our findings suggest that CR orchestrates a coordinated response in the lacrimal gland and on the ocular surface by modulating metabolic pathways, reducing inflammation and immune cell presence, protecting goblet cell density and the corneal barrier, and preserving or enhancing mitochondrial activity and, therefore, LG secretory function. Our findings strongly indicate that CR offers potential therapeutic avenues for age- and inflammation-related gland dysfunction.

One limitation of this work is the relatively small number of biological replicates in our bulk RNA-seq experiment. Notably, one sample in the CR group exhibited an intermediate expression pattern on the heatmap, suggesting it may have been an outlier. This variability in response to CR may reflect inherent biological variability associated with aging [71]. Another limitation of our study was that evaluation of macrophages infiltrating the LGs could only be performed qualitatively in immunofluorescent images without standardized image analysis (Fig. 5G) due to the small number of biological replicates collected for histology. In future studies, we plan to address this quantitatively by conducting flow cytometry analysis of CR LGs. We have used flow cytometry to quantify immune cell infiltrations within the LGs [54, 72]. Additionally, our study exclusively used female mice. Since it is well known that sex influences immune responses, metabolic pathways, and aging processes, it would be important to investigate whether CR elicits similar benefits in male mice. Indeed, sex-specific responses to CR have been found in other tissues, like the liver and adipose tissue, due to putative hormonal influences [73, 74]. Although the data obtained in this study provide evidence for CR-mediated benefits for female mice, future studies should investigate whether sex-specific factors affect the degree or mechanisms of improvement in LG function.

## Supplementary Information

Below is the link to the electronic supplementary material.

Supplemental Fig. 1 Body mass measurements from mice receiving AL or CR diet during the experiment. (PNG 57.0 KB)

High Resolution Image (TIF 114 KB)

Supplemental Fig. 2 qPCR validation of genes involved in pathways “Salivary secretion” (A) and “Regulation of lipid metabolic process” (B). Each dot represents one lacrimal gland. The Mann-Whitney *U*-test was used. *P* value as shown. (PNG 70.6 KB)

High Resolution Image (TIF 2.76 MB)

Supplementary File 1 List of significant DEGs between CR and AL mice LGs. (XLSX 139 KB)

Supplementary File 2 Log2 fold change of significant DEGs involved in pathways related to Figs. 3 and 4. (XLSX 25.8 KB)

Supplementary File 3 Metascape analysis of upregulated DEGs. (XLSX 97.5 KB)

Supplementary File 4 Metascape analysis of downregulated DEGs. (XLSX 83.4 KB)

Supplementary File 5 List of DEGs in cornea after CR. (XLSX 80.8 KB)

Supplementary File 6 List of DEGs in conjunctiva after CR. (XLSX 4.28 MB)
