# Supplementary figures and images for: Exploring the transformative effects of calorie restriction on the lacrimal gland in adult mice
Source: GeroScience. 2025 Jun 28;47(5):6157–76. doi: 10.1007/s11357-025-01748-w (PMC12634954; doi:10.1007/s11357-025-01748-w)

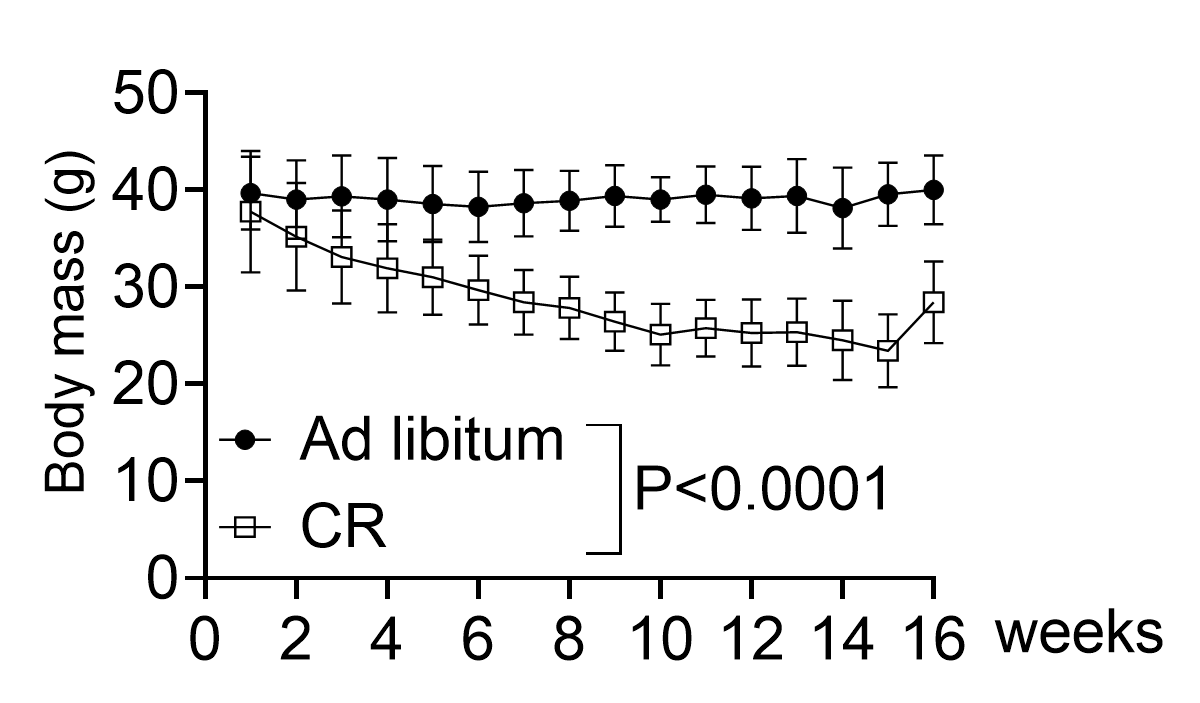

Supplement: Supplementary file 1 — Body mass measurements from mice receiving AL or CR diet during the experiment. (PNG 57.0 KB) [file 11357_2025_1748_Fig9_ESM.png]

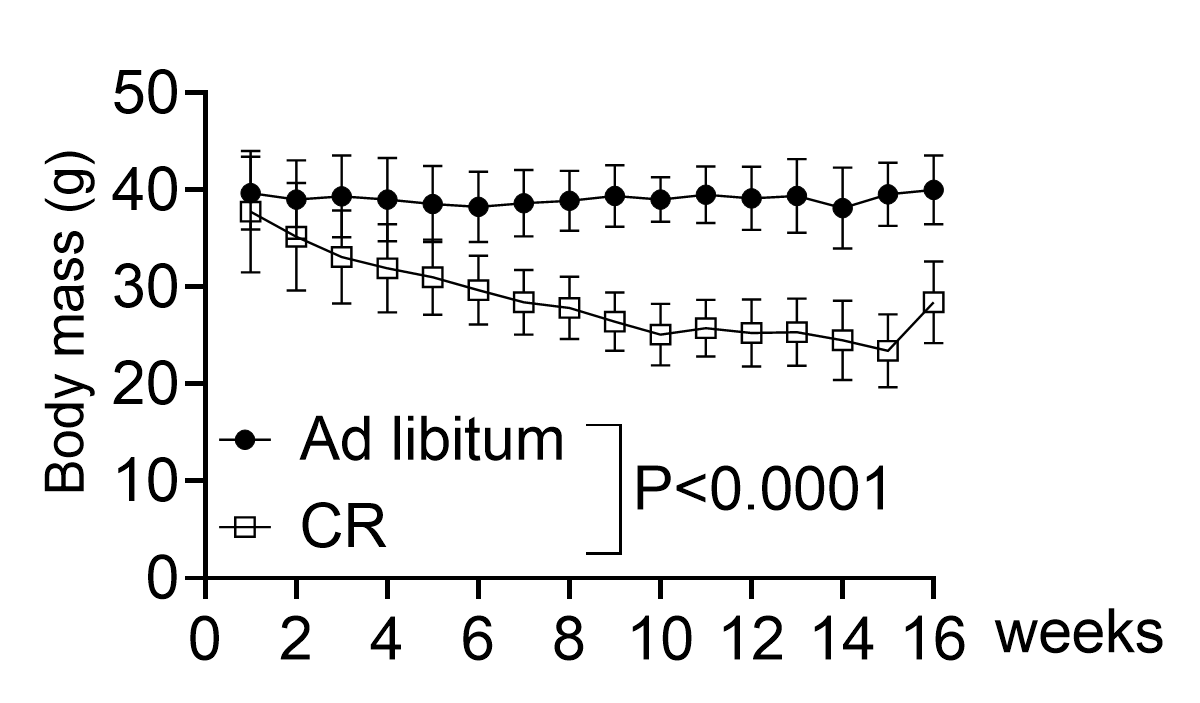

Supplement: Supplementary file 2 — High Resolution Image (TIF 114 KB) [file 11357_2025_1748_MOESM1_ESM.tif]

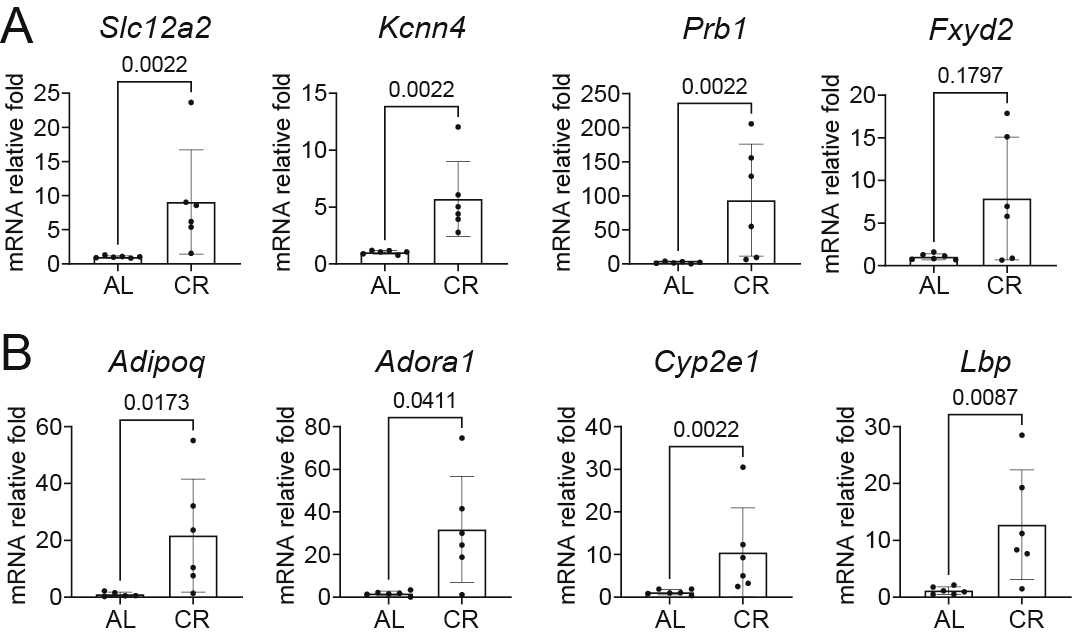

Supplement: Supplementary file 3 — qPCR validation of genes involved in pathways “Salivary secretion” (A) and “Regulation of lipid metabolic process” (B). Each dot represents one lacrimal gland. The Mann-Whitney U-test was used. P value as shown. (PNG 70.6 KB) [file 11357_2025_1748_Fig10_ESM.png]

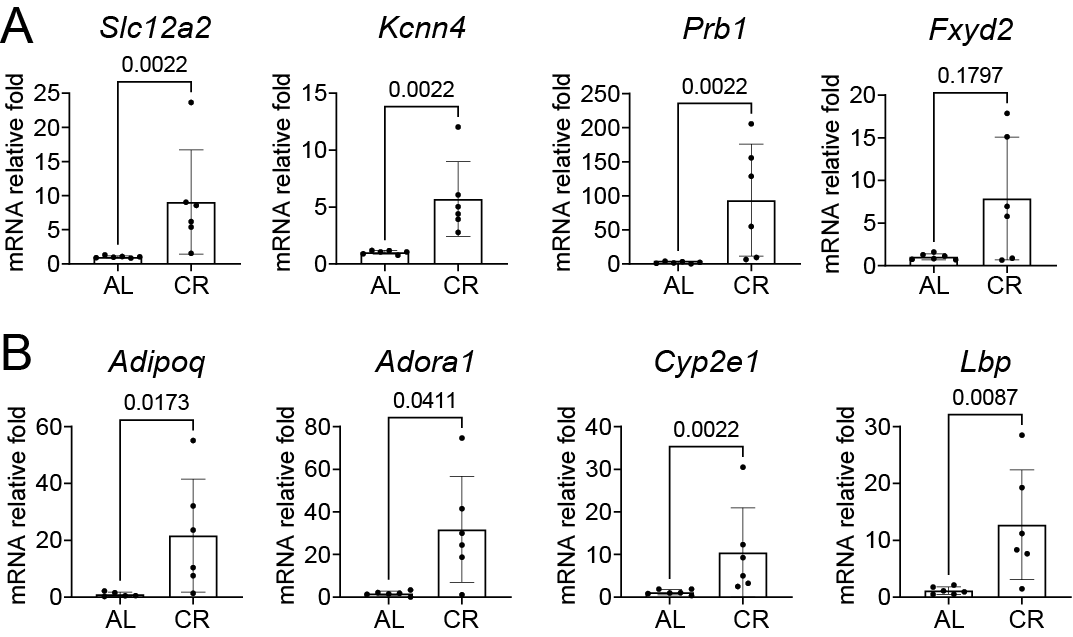

Supplement: Supplementary file 4 — High Resolution Image (TIF 2.76 MB) [file 11357_2025_1748_MOESM2_ESM.tif]
